# Current outcomes and treatment of tetralogy of Fallot

**DOI:** 10.12688/f1000research.17174.1

**Published:** 2019-08-29

**Authors:** Jelle P.G. van der Ven, Eva van den Bosch, Ad J.C.C. Bogers, Willem A. Helbing

**Affiliations:** 1 Department of Pediatrics, Division of Pediatric Cardiology, Erasmus MC-Sophia Children’s Hospital, Rotterdam, The Netherlands; 2 Netherlands Heart Institute, Utrecht, The Netherlands; 3 Department of Cardiothoracic Surgery, Erasmus MC, Rotterdam, The Netherlands; 4 Department of Pediatrics, Division of Pediatric Cardiology, Radboud UMC - Amalia Children's Hospital, Nijmegen, The Netherlands

**Keywords:** Tetralogy, Fallot, Congenital Heart Disease, Survival, Outcomes

## Abstract

Tetralogy of Fallot (ToF) is the most common type of cyanotic congenital heart disease. Since the first surgical repair in 1954, treatment has continuously improved. The treatment strategies currently used in the treatment of ToF result in excellent long-term survival (30 year survival ranges from 68.5% to 90.5%). However, residual problems such as right ventricular outflow tract obstruction, pulmonary regurgitation, and (ventricular) arrhythmia are common and often require re-interventions. Right ventricular dysfunction can be seen following longstanding pulmonary regurgitation and/or stenosis. Performing pulmonary valve replacement or relief of pulmonary stenosis before irreversible right ventricular dysfunction occurs is important, but determining the optimal timing of pulmonary valve replacement is challenging for several reasons. The biological mechanisms underlying dysfunction of the right ventricle as seen in longstanding pulmonary regurgitation are poorly understood. Different methods of assessing the right ventricle are used to predict impending dysfunction. The atrioventricular, ventriculo-arterial and interventricular interactions of the right ventricle play an important role in right ventricle performance, but are not fully elucidated. In this review we present a brief overview of the history of ToF, describe the treatment strategies currently used, and outline the long-term survival, residual lesions, and re-interventions following repair. We discuss important remaining challenges and present the current state of the art regarding these challenges.

## Introduction

**Figure 1.**
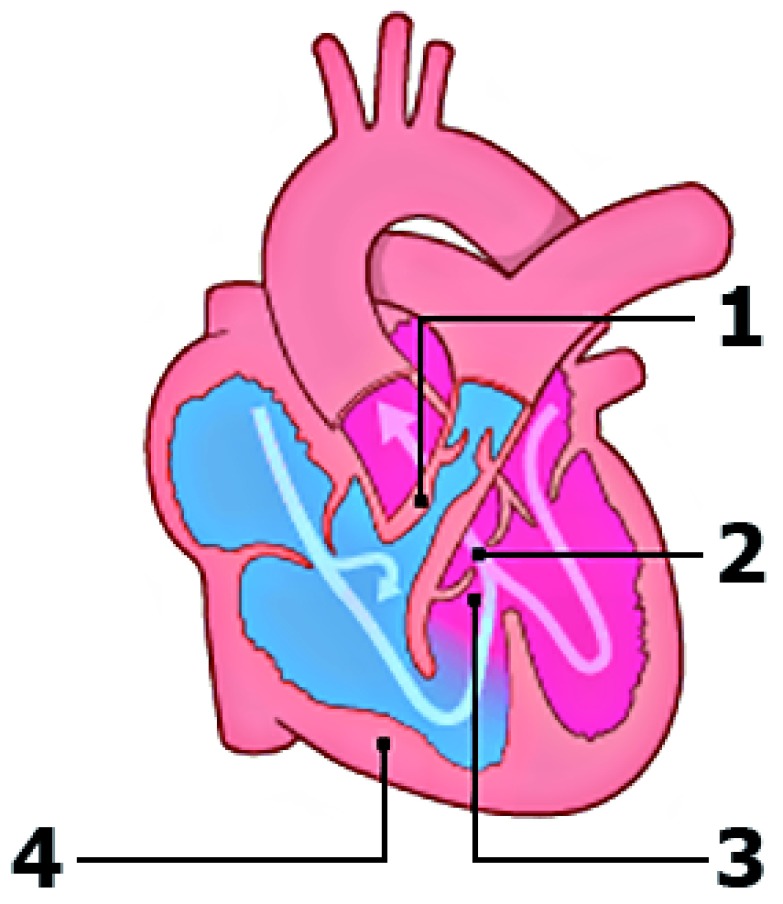
Schematic overview of the defects seen in tetralogy of Fallot. (1) Pulmonary stenosis. (2) Overriding aorta. (3) Malalignment ventricular septal defect. (4) Right ventricular hypertrophy. Modified from Englert *et al*. ^4^ with permission from the publisher.

Tetralogy of Fallot (ToF), the most common type of cyanotic congenital heart disease (CHD), has an incidence of 0.34 per 1000 live births ^1^. The classic tetrad ( Figure 1) was first described in 1673 by bishop and anatomist Nicolas Steno, but the anatomy was more extensively described by the French physician Étienne-Louis Fallot in 1888 ^2, 3^. Patients with ToF have varying degrees of cyanosis depending on the severity of right ventricular outflow tract (RVOT) stenosis and pulmonary artery (PA) anatomy. The anatomic abnormalities seen in ToF vary from milder to more severe phenotypes, such as ToF with pulmonary atresia and Fallot-type double outlet right ventricle (RV). These more severe forms may require different management and treatment strategies. This review focuses on the “classic” ToF, with right ventricular outflow (pulmonary) stenosis, rather than atresia, and excluding double outlet right ventricle.

## Surgical approaches to repair

**Figure 2.**
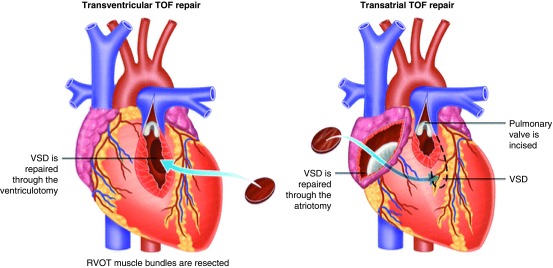
Transventricular (left) and transatrial-transpulmonary (right) approach to tetralogy of Fallot (ToF) repair. VSD, ventricular septal defect. Adapted from Bushman ^18^ with permission from the publisher.

Surgical repair of ToF was first described in 1955 by Lillehei *et al*. ^5^. The right ventricular outflow tract obstruction (RVOTO) was approached by a ventriculotomy into the right ventricular anterior wall and relief included inserting a transannular patch (TAP) if required ( Figure 2, left). Aggressive RVOTO relief was advocated as initial results had demonstrated that residual RVOTO was predictive of early mortality ^6^. This approach resulted in relatively good long-term survival ^7^. However, residual lesions after repair were common and follow-up studies of these first operations showed that these residual lesions resulted in late morbidity and mortality ^8– 11^. Pulmonary regurgitation (PR) was reported in the majority of patients, more commonly in those with TAPs ^12^. PR initially was thought to be a relatively benign hemodynamic residual lesion but subsequently was found to be predictive of decreased exercise performance and progressive RV dilation. RV dilation, in turn, was associated with ventricular arrhythmia and biventricular dysfunction ^13– 15^. Furthermore, patients were noted to be at higher risk of sudden cardiac death ^8, 9, 11, 16, 17^.

Different surgical techniques were developed minimizing the extent of the ventriculotomy and trying to preserve competence of the pulmonary valve without causing significant residual RVOTO. Via a transatrial or transatrial-transpulmonary approach, the need for a ventriculotomy can be reduced ( Figure 2, right). The transatrial or transatrial-transpulmonary approach is currently employed in most centers, and the long-term results are excellent ^12, 19– 22^. In patients with a small pulmonary valve annulus, a TAP is still necessary for adequate RVOTO relief. ** Other techniques to preserve or replace pulmonary valve competence include pulmonary valvuloplasty with patching limited to the infundibulum ^23, 24^, implantation of a monocusp valve ^25, 26^, a valved RV-to-PA conduit ^27, 28^, or a homograft valve ^27^. A survival benefit of these valve-sparing or valve-replacing techniques has not yet been demonstrated ^29– 32^.

## Variations in current treatment strategies

In general, it is thought that earlier primary repair of ToF can limit prolonged exposure to RV pressure loading and reduced oxygen saturations, preserving cardiovascular ^33^ and brain ^34^ function. However, there is no consensus on the definition of “early” versus later repair. Neonatal repair (that is, repair before 1 month of age) is feasible with acceptable results but is not widely used and this is because of better short-term outcomes of non-neonatal repair ^35^. Neonatal repair more often requires TAP compared with repair beyond the neonatal period, resulting in worse event-free survival ^35^. In the majority of patients, primary repair can be postponed to 3 to 6 months of age with excellent outcomes ^36, 37^.

Symptomatic ToF patients may require an intervention in the neonatal period. Different strategies can be used if primary repair is judged not to be the best option. Historically, a systemic-to-pulmonary shunt—typically a modified Blalock-Taussig (mBT) shunt—has been used to increase pulmonary flow, reduce hypoxemia, and allow time for PA growth. This allows repair to be performed at an older age and has the potential advantage of using no, or less extensive, TAP. However, palliative shunt procedures are associated with a 3% to 5% early mortality rate ^38, 39^. The superiority of a staged approach versus primary neonatal repair has not been demonstrated ^40, 41^.

Stenting of the ductus arteriosus (DA) is another strategy to warrant pulmonary blood flow after birth by inducing a systemic-to-pulmonary shunt. However, in cyanotic CHD, the anatomy of the DA might be complex and unsuited for stenting ^42^. Procedural success is estimated to be 83% ^43^. Recently published multicenter studies compared outcomes following DA stenting and mBT shunting using propensity score–adjusted models ^43, 44^. Clinical status, assessed by saturation, hemoglobin levels, and PA size, was more favorable following DA stenting compared with mBT shunting ^43, 44^. Bentham *et al*. found better survival (hazard ratio 0.25, 95% confidence interval (CI) 0.07–0.85) for DA stent compared with mBT ^43^, whereas Glatz *et al*. found no difference in survival (hazard ratio 0.64, 95% CI 0.28–1.47) ^44^. A trend toward higher re-intervention rate in the DA stent group was observed in both studies ^43, 44^. DA stenting appears to be a feasible strategy for selected cases.

Alternatively, palliative balloon dilation of the pulmonary annulus can be used to increase oxygen saturation and promote growth of the pulmonary vasculature and as bridge to later complete repair in selected patients ^45, 46^. Whether this strategy ultimately reduces TAP use or improves long-term outcomes remains controversial ^45, 46^.

Similarly, RVOT stenting can be used as a palliative strategy or bridge to repair in neonatal life ^47, 48^. Experience with this strategy is still relatively limited but it has been demonstrated to be a relatively safe procedure promoting growth of the pulmonary arteries as a bridge to repair ^48– 50^. Quandt *et al*. compared medium-term outcomes of RVOT stent with systemic-to-pulmonary shunt and found no difference in survival between strategies ^49^. Intensive care and hospital stay duration and peri-operative complications were more favorable for the RVOT stenting group but the re-intervention rate was higher for this group ^49^. The most common re-interventions in this group were re-stenting and re-ballooning. (Re)shunt surgery or early complete repair was less common in this group compared with patients who underwent primary mBT. Comparisons between neonatal repair and RVOT stenting have shown comparable short-term and long-term outcomes ^51, 52^. During 10 years of follow-up, Wilder *et al*. demonstrated a similar increased rate of catheter-based re-interventions in the RVOT stent group compared with neonatal repair ^52^. More studies are needed to determine the best strategy for the patient group requiring early intervention. Management strategies likely need to be individualized for optimal outcome.

## Overall survival

**Figure 3.**
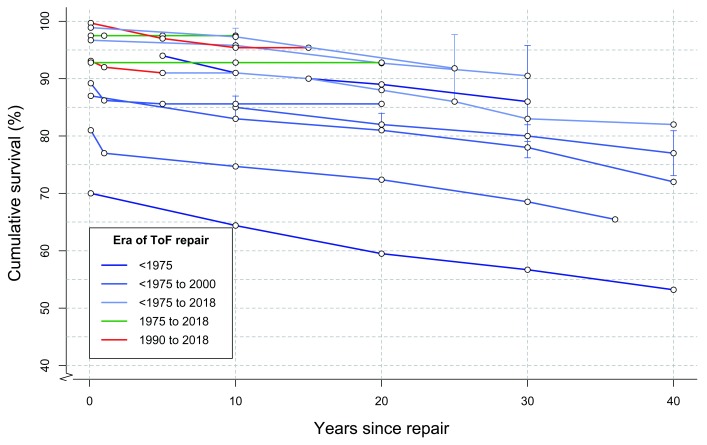
Survival following tetralogy of Fallot (ToF) repair. Each colored line represents a single study, and dots represent Kaplan–Meier survival estimates at different time points ^12, 53– 65^. Ninety-five percent confidence intervals, where published, are shown in vertical lines. Lines are colored according to surgical era.

Overall survival following ToF repair has significantly improved in recent eras. Figure 3 outlines survival in several large studies published within the last two decades, and follow-up was up to 40 years for older cohorts ^12, 53– 65^. Early mortality has significantly decreased in more recent eras. European and American congenital cardiothoracic surgery registries have reported a peri-operative mortality below 3% in recent years ^66– 68^. Peri-operative outcomes are determined largely by the severity of the ToF described by, for example, the pre-operative size of the pulmonary valve and pulmonary arteries, RV-PA pressure gradient, and oxygen saturation ^61, 69– 71^. Patients with repair including TAP have higher peri-operative mortality ^66^. As most centers consider a TAP only when the pulmonary annulus z-score is lower than −2 or −3, this in part reflects more severe ToF ^21, 72^. Furthermore, co-morbidities, such as coronary abnormalities, prematurity, small body size–associated lesions, and genetic abnormalities, have been associated with increased peri-operative mortality ^61, 69– 71, 73^.

Mortality rates at medium-term follow-up have not changed much across the different surgical eras ( Figure 3) ^65^. Survival at 30 years ranges from 68.5% to 90.5% ^54, 57, 58, 62– 65^. Long-term (20 to 30 years) survival from large cohorts of patients operated on with more recent surgical modifications of ToF repair (for example, valve-sparing and valve-replacing techniques) is still lacking. Important factors determining long-term outcome are residual RVOTO and severity of PR ^54^.

Survival into adulthood is currently expected following ToF repair, leading to a growing population of adults with corrected ToF who require lifelong specialized medical care ^74– 77^. Re-interventions are common in these patients. Cuypers *et al*. found that 44% of patients underwent at least one surgical or catheter re-intervention after 35 years of follow-up ^63^. D’Udekem *et al*. found that 24 ± 5% of patients underwent re-operation after 30 years of follow-up ^64^. Following transatrial transpulmonary repair, lower rates of re-interventions have been reported. Luijten *et al*. ^12^ found a 80% freedom of re-intervention and death after 10 years and D’Udekem *et al*. ^64^ found 75% freedom of re-operation after 25 years. A small case-control study found a lower pulmonary valve replacement (PVR) rate following transatrial repair compared with transventricular repair. The use of a TAP is associated with a higher re-intervention rate ^12, 54^, as is severity of ToF at repair ^56, 65^. Specific indications for re-interventions will be discussed later in this article.

## Residual problems and re-interventions

### Residual right ventricle outflow tract obstruction

Residual RVOTO is common following repair and results in residual or progressive concentric hypertrophy of the RV. Data obtained from the INDICATOR study suggest that RV hypertrophy, due to increased mass-to-volume ratio, is a more important long-term risk factor for ventricular tachycardia (VT) and death than severity of RV dilation (RV end-diastolic volume index) ^78^. Current guidelines provide clear indications for re-intervention for residual RVOTO ( Table 1) ^75– 77^. Balloon valvuloplasty or PVR can be performed for valvular pulmonary stenosis (PS). PA branch stenosis can be safely relieved by balloon dilation, stenting, or PA reconstruction ^79^. In several large studies, 1% to 7% of patients have undergone PA dilation or stenting at long-term follow-up (median of 5.8 to 36 years) ^61, 63, 64, 80, 81^. Surgical relief of the RVOT and PA plasties were performed in 1% to 5% of patients at long-term follow-up ^61, 63, 80, 81^.

**Table 1.** Indications for pulmonary valve replacement in current guidelines.

	European Society of Cardiology (2010) ^77^	American College of Cardiology/American Heart Association (2008) ^75^	Canadian Cardiovascular Society (2009) ^76^
Class I	Symptomatic patients with severe PR and/or PS (RV systolic pressure >60 mm Hg, TR velocity >3.5 m/s)	Severe PR and Symptoms or decreased exercise tolerance	
Class IIa	Severe PR or PS (or both) and either:	Severe PR and either:	Free PR and either:
RV size		Moderate to severe RV enlargement	EDVi 170 mL/m ^2^
Progression of RV size	Progressive RV dilation		Progressive RV dilation
RV function	Progressive RV dysfunction	Moderate to severe RV dysfunction	Moderate to severe RV dysfunction
TR	Progressive TR, at least moderate	Moderate to severe TR	Important TR
PS	PS RV systolic pressure greater than 80 mm Hg, TR velocity 4.3 m/s	Peak instantaneous echocardiography gradient greater than 50 mm Hg or RV/LV pressure ratio greater than 0.7 or Residual RVOT obstruction (valvular or subvalvular) with progressive and/or severe dilatation of the RV with dysfunction	RV pressure at least 2/3 systemic pressure
Exercise capacity	Decrease in objective exercise capacity		Symptoms such as deteriorating exercise performance
Arrhythmia	Sustained atrial or ventricular arrhythmia	Symptomatic or sustained atrial and/or ventricular arrhythmias	Atrial or ventricular arrhythmia

EDVi, end-diastolic volume index; LV, left ventricle; PR, pulmonary regurgitation; PS, pulmonary stenosis; RV, right ventricle; RVOT, right ventricle outflow tract; TR, tricuspid regurgitation.

### Pulmonary regurgitation

PR is very common at medium- to long-term follow-up. Five to ten years after repair, 40% to 85% of patients have moderate to severe PR ^53, 73, 82– 84^. PR induces RV volume overload of the RV with often progressive RV dilation, which may include the development of tricuspid regurgitation (TR) and RV dysfunction. It is often accompanied by prolongation of the QRS complex, and RV dyssynchrony could contribute to the progression of dysfunction ^85– 87^. There generally is a long period in a compensated state, during which RV function is maintained. In some patients, these compensatory mechanisms fail, leading to progressive RV dysfunction ^85, 86^. The mechanisms of RV adaptation and remodeling, as well as the molecular events contributing to the transition from a compensated to a decompensated state, are still poorly understood. Timely restoration of pulmonary valve competence is considered to halt the progressive adverse RV remodeling resulting in RV dysfunction seen in chronic PR.

Thirty-five years after ToF repair, PVR will have been performed in about 40% of patients ^63, 65, 88^. Staged repair and TAP are risk factors for late PVR ^12, 54, 63, 80^, whereas mild residual PS seems to reduce risk ^89^. As more patients with ToF survive into adulthood, PVRs are increasingly being performed ^90^.

PVR is effective in decreasing RV volumes, reducing TR, decreasing QRS duration, increasing left ventricle (LV) ejection fraction (EF), and improving functional status ^91, 92^. It should be noted that no improvement in survival following PVR compared with medical management has been demonstrated to date ^93, 94^.

Homograft or bioprosthetic valves are currently the preferred valves for PVR ^95^. The current 10-year re-PVR–free survival of ToF patients undergoing homograft PVR ranges from 74% to 89% ^95, 96^.

Tissue-engineered valves with a non-synthetic and non-immunogenic surface have the potential to provide lifelong valve replacement ^97^. *In situ* tissue engineering techniques, in which a decellularized “starter scaffold” of polymers can be used to provide shape and structure to the valve, are of particular interest. This scaffold is infiltrated by endogenous cells to provide a regenerating functional valve. As the scaffold would be non-immunogenic, this could provide a relatively cheap “off the shelf” valve. Current studies evaluating tissue-engineered valves in animals and humans show promising early results ^98^.

Several transcatheter PVR strategies have been developed and are increasingly used in a clinical (trial) setting ^99^. However, clinical experience compared with (surgical) homograft PVR is limited ^99^. Procedural success of transcatheter PVR is generally good (>95%) ^100^. The hazard rate for re-intervention following transcatheter PVR ranges from 0.4% to 5.9% per patient-year ^100^. However, high rates of infective endocarditis during follow-up have been described ^101^. Recent results from the MELODY Registry estimate the infective endocarditis risk to be 2.3% per patient-year ^102^. In comparison, the infective endocarditis risk in surgical PVR has been estimated to be 0.3% per patient-year ^103^. Transcatheter PVR has been shown to increase exercise capacity and quality of life 6 months after the procedure ^104, 105^. Direct comparisons with surgical PVR are still lacking.

### Arrhythmia

***Ventricular tachycardia***. VT is a common arrhythmia in the repaired ToF population. Cuypers *et al*. reported a 5% cumulative incidence of sustained VT after a median of 35 years after ToF repair ^63^ and these figures are similar to those of most reports ^58, 106^. However, cumulative incidences of up to 15% have been reported in some adult populations ^107^. Predictors of sustained VT include higher age, number of prior cardiac surgeries, presence of a TAP, LV diastolic dysfunction, and QRS width ^63, 106– 108^. Most guidelines recommend implantable cardioverter defibrillators (ICDs) for patients who have had sustained VT or cardiac arrest ^76, 77^. ICDs are also employed for primary prevention, although selecting high-risk patients who would benefit from ICD implantation remains challenging ^76, 77^. Pacemaker and ICD prevalences in adult ToF populations both range from 5% to 10% ^63, 107, 109^.

Electrophysiological studies can help to determine the underlying substrate, and radiofrequency ablation can be performed. Ablation of monomorphic VT substrates has excellent short-term outcomes with recurrent VT in 18% of patients after a mean follow-up of 34 months ^110^. Another study found a similar recurrence rate (19%) 10 years after ablation ^111^.

***Supraventricular tachycardia.*** The prevalence or cumulative incidence of supraventricular tachycardia (SVT) in adult patients ranges from 4% to 20% ^107– 109, 112^. In the first 10 to 15 years following ToF repair, SVT is relatively uncommon but the incidence rises steadily after this period ^107^. Intra-atrial re-entrant tachycardia, typically involving the right atrium, is the most common type of SVT in patients with ToF ^107^. Two large studies found that SVT was an independent predictor of death or VT ^78, 108^. Few studies have assessed the efficacy of ablation of atrial arrhythmias in corrected ToF, and long-term follow-up is lacking ^113– 115^.

### Aortopathy

Dilation of the aorta is seen in 12% to 24% of adult patients with ToF ^116– 118^. In patients with aortic dilation, aortic root size seems to progressively increase over a period of years. Aortic dissection following ToF appears to be a rare complication ^119^. A population-based study in Texas demonstrated no increased risk for thoracic aortic dissection for patients with ToF compared with the general population ^119^. However, progressive aortic root dilation can lead to malcoaptation of the aortic valve and aortic regurgitation. Furthermore, the elasticity of the dilated aortic root was shown to be reduced in patients with ToF, possibly hampering circulatory function ^120^. The importance of aortopathy in circulatory function and mortality remains incompletely understood.

## Knowledge gaps

### Right ventricular adaptation and remodeling

The mechanisms of RV adaptation and remodeling in response to chronic RV volume overload, resulting from PR, are poorly understood ^121^. In young pig models, chronic PR affects biventricular systolic function, RV myocardial contractility, and LV diastolic performance ^122^. Histopathology of several animal models displays early hypertrophy of the chronically volume-loaded RV and, in a later stage, myocardial fibrosis ^121^. The molecular responses to increased volume or pressure loading of the RV are different from those in the LV ^121, 123– 125^. In a pig model of repaired ToF with induced PR, PS, and an RVOT scar, RV hypertrophy and dilation were found after 23 weeks. The myocardium was characterized by increased collagen deposition, leading to decreased impulse conduction velocity and dispersion ^126^. Similar findings were found in the LV, despite preserved LV function at this stage. This demonstrates biventricular adverse effects are present early in the adverse remodeling process ^127^.

Basic research into RV remodeling has focused mainly on the response to increased pressure loading rather than the predominantly volume-loaded RV as seen in PR ^124, 125^. Volume loading and pressure loading increase myocardial metabolic demand. This metabolic stress induces an increased amount of reactive oxygen species. Compensatory anti-oxidant production in the RV is impaired compared with the LV ^125^. This might imply that the RV is more vulnerable to oxidative stress, as seen in abnormal loading conditions.

In volume-loaded RV mouse models, a clinical course similar to RV dysfunction with volume-loaded RV in humans is observed. RV function is maintained during a compensated phase, followed by RV dysfunction ^128^. Gene expression patterns of the cardiomyocyte in the compensated state differ from those of healthy controls. Several molecular pathways, such as transforming growth factor beta (TGF-β) signaling, p53 signaling, and cytoskeleton-related pathways, are downregulated in the early compensated state but show late upregulation as the RV progressively remodels ^128^. However, the exact cellular and molecular mechanisms of transition from a compensated to a decompensated state of the volume-loaded RV have not been fully elucidated ^125, 129^.

### Assessing the right ventricle in patients with tetralogy of Fallot

Our limited understanding of the pathophysiology of RV failure hampers our ability to adequately detect failure in the early stages in clinical practice. Imaging techniques are used to assess the RV and follow patients serially, aiming to detect early changes in biventricular size and performance. Cardiovascular magnetic resonance (CMR) imaging is routinely used to reliably quantify RV volumes and function, wall mass, and PR ^130^. Adverse clinical events have been related to larger RV volumes, PR severity, biventricular EF, and mass-to-volume ratio ^78, 131, 132^. Increased RV volumes, most commonly end-diastolic volume index (EDVi), have been considered a sign of prolonged high PR burden and thus a predictor of RV dysfunction. However, exercise capacity can be preserved even in severely dilated ventricles, demonstrating that compensatory mechanisms can still be adequate to maintain performance of large RVs ^133^. In the INDICATOR cohort, increased RV wall mass-to-volume ratio, among other factors, was found to be an independent predictor of VT and all-cause mortality, whereas RV EDV and end-systolic volume were not predictive of the end-points ^78^. RV hypertrophy could be a more sensitive marker of pending dysfunction than EDV, although this might be particularly true for patients with residual PS.

Regional myocardial performance and mechanical synchrony can be assessed by strain imaging studies. Global circumferential or longitudinal strain has been used to assess RV function. Under normal circumstances, the RV ejects mainly by longitudinal shortening while, with increased RV pressure loading, circumferential contraction is increased ^134^. The predictive value of global longitudinal or circumferential strain in ToF is still uncertain: Orwat *et al*. found that RV global longitudinal strain assessed by CMR was a superior independent predictor for death, cardiac arrest, or VT compared with RV volumes ^135^. RV global circumferential strain was not predictive of outcome in that study ^135^. Diller *et al*. found a similar relation for LV global longitudinal strain assessed by echocardiography ^136^.

Mechanical dyssynchrony has been demonstrated to relate to prolonged or fragmented (QRS complex containing additional spikes without bundle branch block) QRS complexes ^137^. The contributions of this mechano-electrical interaction to RV function remain uncertain, as studies assessing mechanical dyssynchrony report conflicting results ^135, 136, 138– 141^. RV circumferential dyssynchrony was shown to negatively predict exercise capacity in one study ^140^. This association has not been confirmed in other studies ^138, 141^. Cardiac resynchronization therapy is increasingly used in ToF. A recent study found that 12 out of 15 adult patients with ToF had an improved NYHA (New York Heart Association) class or LV function after 2.6 years (median) of cardiac resynchronization therapy ^142^. Procedural success was high and adverse events were rare.

### Right ventricular interactions in tetralogy of Fallot

***Atrio-ventricular interactions.*** Diastolic function after ToF repair is a determinant of the amount of PR. In some patients, end-diastolic forward flow (EDFF) in the main PA during right atrial contraction can be observed ^143^. This is considered a sign of “restrictive RV physiology” as the non-compliant RV acts as a conduit during atrial contraction as RV diastolic pressure exceeds PA diastolic pressure ^144, 145^. Restrictive physiology could limit the amount of PR as elevated diastolic RV pressure reduces the amount of PR. A recent study found no relationship between the presence of EDFF and other markers of diastolic dysfunction (that is, RV hypertrophy, atrial dilatation, reduced stroke volume, or reduced PR) ^146^. Different mechanisms, such as pulmonary arterial capacitance and atrial function ^147^, may play significant roles in the occurrence of EDFF. Luijnenburg *et al*. found that bi-atrial function, but not diastolic ventricular function, differed between patients with EDFF and those without it ^147^. In that study, abnormal atrial function was related to worse exercise capacity and higher N-terminal pro brain natriuretic peptide (NT-proBNP). Kutty *et al*. found that right atrial longitudinal strain predicted RV performance but not exercise capacity ^148^.

The effect of EDFF on circulatory function is controversial. Studies found conflicting results regarding the relationship between EDFF and the amount of PR ^143, 144, 146^, exercise capacity ^144– 147^, and EDV ^144– 147^. The presence of EDFF might have a different etiology and clinical importance early versus late after repair or in severely dilated versus non-dilated ventricles.

***Ventriculo-arterial interactions.*** Adequate atrio-ventricular coupling and ventriculo-arterial (VA) coupling are required for an energetically efficient transfer of blood through the right heart. VA coupling has not been studied extensively in ToF. Latus *et al*. assessed VA coupling as the relationship between pulmonary arterial elastance and ventricular end-systolic elastance in adult patients with ToF by using CMR and catheter-derived measurements both in resting conditions and during dobutamine stress ^149^. VA coupling was impaired during resting conditions. EF and load-independent parameters of RV contractility increased during dobutamine stress. Pulmonary arterial elastance increased accordingly and the impaired VA coupling that resulted during dobutamine stress was similar to that under resting conditions.

***Interventricular interactions.*** Interactions between the RV and LV have been extensively described. The LV and RV have common myocardial fibers, the interventricular septum, the anatomic space confined by the pericardium, and a common neurohumoral system ^150^. Not unexpectedly, the effects of chronic PR are not limited to the RV, although the mechanisms of this ventriculo–ventriculo interaction in chronic PR remain poorly understood. A linear correlation between LV and RV EF has been described ^150, 151^. Severe RV dilation causes abnormal diastolic septal positioning, influencing LV filling ^152^. The role of the LV in outcomes in ToF is increasingly appreciated, as LV function has been associated with increased mortality and increased risk of VT ^136, 153^. In the INDICATOR registry, LV EF was one of three independent predictors of mortality and VT ^154^. Geva *et al*. found that LV EF, independent of RV parameters, predicted poor functional status ^151^. Remarkably, parameters of LV function are not considered in current guidelines for the timing of PVR ( Table 1).

### Drug therapy for right ventricular failure

Pharmacotherapy is important in the treatment of LV failure and improves outcomes. However, the effects of the use of heart failure medication for RV failure have been disappointing ^155– 157^. In patients after ToF repair, RAAS (renin–angiotensin–aldosterone system) inhibitors do not appear to influence RV EF or exercise capacity ^158^. In a randomized controlled trial of 33 patients with ToF, beta blockers showed no beneficial effects after 6 months of treatment and an increase in NT-proBNP was noted ^159^. Increasing our understanding of the pathophysiology of RV failure might elucidate new targets for medical treatment unique to the RV.

### Current guidelines on the timing of pulmonary valve replacement

Restoring pulmonary valve function before irreversible RV dysfunction occurs could be important to prevent RV failure. However, the durability of currently used pulmonary prosthetic valves is limited. Therefore, the timing of PVR always is a compromise: It should be timed early enough to prevent irreversible adverse remodeling but late enough to limit the number of re-interventions. Because of the difficulties in assessing RV function, predicting decline in RV function is difficult, and the optimal timing of PVR is controversial. Guidelines by the European Society of Cardiology, the Canadian Cardiovascular Society (CCS), and the American College of Cardiology/American Heart Association provide some recommendations on indications for performing PVR ^75– 77^. These indications are summarized in Table 1.

Indications differ between guidelines and have several limitations. Most guidelines do not provide specific cutoff points since these are statistical constructs that do not work for individual patients. The 2009 CCS guideline provides an absolute cutoff value for EDVi but does not take into account the considerable differences in normal (indexed) RV volumes between genders and age ^160^. End-systolic volume index and RV mass-to-volume ratio have been proposed as superior predictors compared with EDV ^78, 161^. Progressive RV dilation is considered an indication for PVR, but there is no consensus on what too much progression is ^162– 165^. Longitudinal changes in RV size and function following ToF repair have been reported in several studies ^166– 173^. RV volumes increase non-linearly and seem to stabilize in adolescence. These factors need to be taken into account when assessing progressive RV dilation.

Furthermore, the recommendations in current guidelines are often based on long-term outcomes of studies in patients who have been operated at a much older age than has been the practice in the past 20 years. This warrants caution when extrapolating these results to current adolescent or younger patients.

Careful interpretation of current guidelines seems to be justified. Individual patient parameters and views should always be taken into consideration. In clinical practice, an approach using information from different sources, including history, physical examination, electrocardiogram, imaging techniques, exercise testing, and blood biomarkers, may be most useful ^174^.

## Conclusions

ToF can be repaired with low short-term and long-term mortality. This has caused a demographic shift such that many patients survive well into adulthood. Long-term follow-up of older cohorts has shown the detrimental effects of PR in the long-term. However, residual lesions cause significant morbidity. Surgical modifications to preserve pulmonary valve function, such as the transatrial (and transpulmonary) approaches and restricted use of TAPs, have been widely adopted. Despite improvements in morbidity, follow-up duration for these techniques is probably too limited to demonstrate a survival benefit.

Our limited understanding of RV adaptation and the pathophysiology of RV heart failure hampers the ability to detect failure in early stages in clinical practice and to predict future decline of RV function. While a large proportion of adult ToF survivors require one or multiple PVRs in their lifetimes, selecting optimal candidates and optimal timing for PVR remains challenging. Increasing our understanding of RV failure seems key to answer these difficult questions. This might provide treatment options to attain optimal long-term health outcomes for patients with ToF.

## Abbreviations

CCS, Canadian Cardiovascular Society; CHD, congenital heart disease; CMR, cardiovascular magnetic resonance; DA, ductus arteriosus; EDFF, end-diastolic forward flow; EDV, end-diastolic volume; EDVi, end-diastolic volume index; EF, ejection fraction; ICD, implantable cardioverter defibrillator; LV, left ventricle; mBT, modified Blalock-Taussig; NT-proBNP, N-terminal pro brain natriuretic peptide; PA, pulmonary artery; PR, pulmonary regurgitation; PS, pulmonary stenosis; PVR, pulmonary valve replacement; RV, right ventricle; RVOT, right ventricular outflow tract; RVOTO, right ventricular outflow tract obstruction; SVT, supraventricular tachycardia; TAP, transannular patch; ToF, tetralogy of Fallot; TR, tricuspid regurgitation; VA, ventriculo-arterial; VT, ventricular tachycardia
